# Cosmetic Surgery and Self-esteem in South Korea: A Systematic Review and Meta-analysis

**DOI:** 10.1007/s00266-019-01515-1

**Published:** 2019-10-21

**Authors:** Sanghoo Yoon, Young A. Kim

**Affiliations:** 1 Division of Mathematics and Big Data Science, Daegu University, Gyeongsan-si, Gyeongsanbuk-do Republic of Korea; 2 College of Nursing, Jeju National University, 102 Jejudaehakno, Jeju-si, Jeju-do 63243 Republic of Korea

**Keywords:** Esthetic, Cosmetic, Plastic surgery, Attitude, Self-esteem

## Abstract

**Purpose:**

Advances in medical technology coupled with rapid growth of web-based mass media and social networking services have considerably increased public access to cosmetic surgery. In South Korea, in particular, the number of people undergoing cosmetic surgery has been rapidly increasing, and studies related to cosmetic surgery have markedly increased. We report an integrative review of studies examining the relationship between cosmetic surgery and self-esteem in Korea. We aimed to identify relevant variables and determine their overall effect sizes.

**Methods:**

This study was designed based on the Preferred Reporting Items for Systematic reviews and Meta-Analyses guidelines. Two researchers separately performed the literature search, selected 16 papers based on the inclusion and exclusion criteria, and analyzed them.

**Results:**

Of the 16 papers on cosmetic surgery and self-esteem, 5 (33.3%) involved both men and women, and the remaining 11 (66.7%) involved only women. The respondents included teenagers and adults. The total number of respondents was 6296, with an average of 393.5 per paper. Most studies (*n* = 13, 81.3%) used the Rosenberg Self-Esteem Scale. Self-esteem was correlated with variables grouped into the following six categories: appearance management intention, cosmetic surgery intention, sociocultural attitude, body satisfaction, BMI, and stress. The effect sizes from the meta-analysis with correlation coefficients were 0.157, − 0.118, 0.023, 0.175, − 0.045, and − 0.085.

**Conclusions:**

Among the relevant variables categorized in this study, sociocultural attitude, BMI, and stress showed weak effect sizes, and the appearance management intention, cosmetic surgery intention, and body satisfaction categories showed intermediate effect sizes. The results of this study are expected to serve as a concrete basis for the development of strategies to minimize the adverse effects of the ever-growing cosmetic surgery industry. This information can help elucidate the psychologic characteristics of individuals seeking cosmetic surgery and contribute to optimal medical outcomes.

**Level of Evidence IV:**

This journal requires that authors assign a level of evidence to each article. For a full description of these Evidence-Based Medicine ratings, please refer to the Table of Contents or the online Instructions to Authors www.springer.com/00266.

## Introduction

### Rationale for the Study

Public access to cosmetic surgeries has greatly increased because of advances in medical technology coupled with rapid growth of web-based mass media and social media. In South Korea (hereafter “Korea”) in particular, an increasing number of people have been undergoing cosmetic surgery. According to the International Society of Aesthetic Plastic Surgery (ISAPS), Korea ranks first on a per capita basis, with 13.5 cosmetic procedures performed per 1000 individuals [1, 2]. However, because the samples of ISAPS studies only include esthetic plastic surgeons worldwide, the actual number of cosmetic procedures is possibly higher if the statistics include those performed by general practitioners. Furthermore, we can infer this number to be even higher given that no official statistics are available in Korea because cosmetic surgeries are not covered by the Korean health insurance system [3]. According to a recent survey conducted in Korea, the proportion of people who have considered but not undergone cosmetic surgery increased from 14% in 1994 to 15% in 2004 and 18% in 2015; similarly, the proportion of people who actually underwent cosmetic surgery increased from 2% in 1994 to 5% in 2004 and 7% in 2015 [4].

With an increasing number of people seeking to undergo cosmetic surgery, research in this field has been rapidly growing. This field shows three main research trends: studies examining the psychopathology of cosmetic surgery consumers [5–7], studies on the desire for cosmetic surgery [8–11], and studies on the influence of mass media or celebrity worship on cosmetic surgery [12–14]. A systematic review and meta-analysis comparing pre- and postoperative quality of life after cosmetic surgery found that the overall quality of life, in both the psychologic and physical domains, improved postoperatively [15].

Interest in cosmetic surgery is associated with body image, self-esteem, individual propensities, and psychologic factors, such as psychopathologic traits [7]. A strong correlation exists between cosmetic surgery and self-esteem, wherein self-esteem increases or decreases depending on the situation [16]. In particular, self-esteem associated with body image has a great influence on choosing the type of procedure [5]. However, a lack of consensus exists among researchers regarding the relationship between cosmetic surgery and self-esteem; some studies reported that self-esteem has little bearing on the level of addiction to cosmetic surgery [10, 17], whereas others reported that low self-esteem influences the choice of procedure and that self-esteem increases after cosmetic surgery [18–21]. Thus, an integrative review of studies examining the relationship between cosmetic surgery and self-esteem is needed. Meta-analyses can serve as powerful tools to derive an integrative result from a set of studies [22]. Korean studies on cosmetic surgery have mostly only focused on the limited aspects of the relationships between cosmetic surgery and mental health [23–25]; thus, a systematic and integrative analysis of variables related to cosmetic surgery is lacking.

### Objectives of the Study

The research question formulated was as follows: What factors found by studies examining the relationship between cosmetic surgery and self-esteem are relevant? To address this question, we performed an integrative review of studies on the relationship between cosmetic surgery and self-esteem. Our aim was to combine the outcomes of individual studies and perform a meta-analysis based on the correlation coefficients (effect sizes) of the major variables in the existing studies to elucidate correlations among them. The results of this study are expected to serve as the foundation to help individuals raise awareness and develop strategies to minimize the adverse effects of increasingly prevalent cosmetic surgeries by focusing on the psychologic factors in individuals seeking these surgeries.

## Methods

### Study Design

This study is a systematic review and meta-analysis based on the Preferred Reporting Items for Systematic reviews and Meta-Analyses guidelines that aims to derive relationship factors from studies on cosmetic surgery and self-esteem [26, 27].

### Data Selection and Collection

To select studies for the meta-analysis, a literature review was performed. Articles for the literature review were selected based on the research question mentioned in the Introduction section. The inclusion criteria were survey-based papers that examined the relationship between cosmetic surgery and self-esteem published in Korean in Korean journals. In terms of the PICO framework [28], the participants (P) were human subjects, interventions (I) and comparisons (C) did not apply because only survey studies were considered, and the outcomes (O) were inter-variable correlations. Therefore, studies without a correlation analysis or correlation coefficients were excluded. Furthermore, studies presenting only the abstract, data analyses, conference papers, dissertations, and qualitative studies were also excluded.

**Fig. 1:**
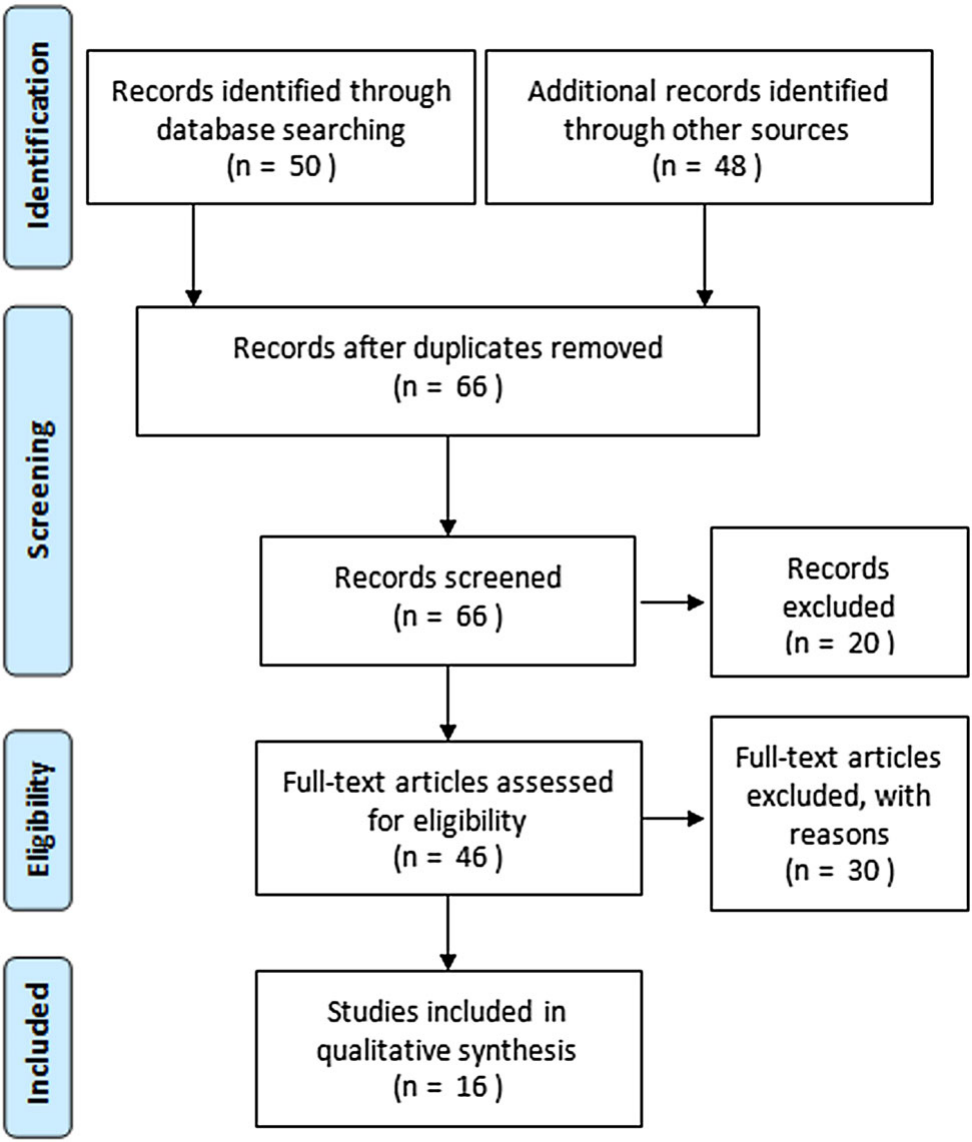
Flow of studies included from the database search

Prior to the literature search, we checked for MeSH terms that applied to a literature search for cosmetic surgery and self-esteem using the PubMed MeSH database. The search period was from the default beginning year to the latest update as of September 2017. The search terms (in Korean) “cosmetic surgery” AND “self-esteem” were entered. Fifty articles were retrieved from the Research Information Service System (RISS) and Korean Studies Information Service System (KISS). Together with 48 additional articles retrieved from Google Scholar and bibliographic cross-referencing, we drafted the first research list of 98 articles. After removing 32 duplicates, we checked the titles and abstracts of the remaining 66 articles and removed an additional 20 articles (14 irrelevant studies, 4 qualitative studies, 1 study with non-human subjects, and 1 data analysis). Then, we performed a full-text check on the remaining 46 articles to determine their academic adequacy based on the inclusion criteria and removed 30 articles meeting the exclusion criteria (25 studies without a correlation analysis and 5 conference papers). Consequently, 16 papers were used for the final analysis. Two researchers involved in nursing and statistics performed the literature search independently. When discrepancies arose over paper choices between the two researchers when applying the inclusion and exclusion criteria, the final selection was made in consensus by reviewing the full text together (Fig. 1).

The methodologic quality of the selected papers was quantitatively assessed by assigning scores (yes, no, or N/A) to the following items using the Critical Review Form [29]: research purpose; literature review; design; sampling; measurement variables; intervention; presentation of statistics and results; conclusion; significance. All 16 papers used for the final analysis were cross-sectional studies that satisfied the requirements of the research purpose, literature review, design, sampling, measurement variables, intervention, presentation of statistics and results, conclusion, and significance items. The sample size calculation process was presented in 3 studies (18.8%); the remaining 13 studies (81.2%) had sample sizes ranging from 156 to 813 subjects, which satisfied the minimum sample size requirement for correlation coefficient calculation. Twelve studies (75.0%) mentioned the survey sample attrition rate. The methodologic quality was assessed by the two researchers independently, followed by a crosscheck; in case of discrepancies, a reassessment was performed after reaching a consensus through a full-text review.

### Data Analysis

Each study was analyzed against an eight-item checklist: age group of the subjects, sex, sample size, sample size calculation, researchers’ academic disciplines, self-esteem measurement tool used, relationship variables, and correlation coefficients. Data coding was performed using MS Excel. For the meta-analysis, the correlation coefficient effect size was calculated with the R program meta package. Inverse variance weighting was used for pooling to calculate the overall effect size with correlation coefficients. Explaining the overall effect size with a meta-regression analysis through covariances is difficult, because several variables can be obtained in a study. Therefore, the results of related homogeneous variables were presented through k-means clustering, which is a method that aims to partition correlation coefficients into k clusters with the nearest mean. This approach was used until the minimum homogeneous cluster was achieved. In addition, random-effects models with inverse variance weights were used without further correction.

## Results

Table 1 presents the analysis of the characteristics of the 16 studies. College students were surveyed most frequently (*n* = 8), followed by high school students (*n* = 4) and adults (*n* = 1); three studies surveyed subjects in all of these age groups. Five studies (33.3%) surveyed both men and women, and the remaining 11 studies (66.7%) surveyed only women. The total number of subjects was 6296 (i.e., 393.5 subjects per study on average). The most frequent single discipline of the researchers was nursing (*n* = 3), followed by apparel science, business administration (*n* = 2 each), cosmetology, education, and psychology (*n* = 1 each). Four studies were interdisciplinary. Most studies (*n* = 13, 81.3%) used the Rosenberg Self-Esteem Scale [46], and a total of 70 variables were identified to be correlated with self-esteem.

**Table 1:** Descriptive summary of selected studies

References	Sample				Researcher’s major	Related variables	Self-esteem measurement
	Age group (mean)	Sex	*n*	Cal.			
Chung and Lee [30]	Undergraduate (unclear)	F	356	–	Fashion design, education	CA (esthetics), CA (body enhancement), CA (celebrity imitation behavior), CA (luxury goods preference)	Rosenberg (1965)
Ha and Kang [31]	High school student (unclear)	F	391	–	Beauty science, education	Appearance MB (AS needs), appearance MB (diet), appearance MB (makeup, hairstyle), stress (relationship with friends), stress (relationship with parents), stress (school records)	Coopersmith (1967)
Hwang et al. [32]	Undergraduate (unclear)	M, F	685	–	Fashion design	CS behavior	Rosenberg (1965)
Jang and Song [33]	High school student (unclear)	M, F	631	–	Education	Appearance satisfaction	Coopersmith (1967)
Jeon and Lee [34]	10–40 s (unclear)	F	813	–	Apparel science	AS conformity, AS cost payment, AS risk tolerance, AS secret, AS value	Rosenberg (1965)
Jeon and Lee [35]	10–40 s (unclear)	F	813	–	Apparel science	AS desire (face), AS desire (lipoplasty), AS desire (tatoo, breast augmentation, breast reduction), CA (esthetics), CA (fashion), CA (sexual attractiveness)	Rosenberg (1965)
Kim [36]	Undergraduate (unclear)	F	156	–	Nursing	AEBI, BAS, CS perception	Rosenberg (1965)
Kim and Kim [37]	High school student (unclear)	M, F	289	–	Beauty science	Fashion MB, hair care behavior, makeup MB, skin care behavior, weight control behavior	Rosenberg (1965), Coopersmith (1967), Pope et al. (1988)
Kim et al. [38]	Undergraduate (21.13 ± 2.12)	M, F	255	+	Nursing	CS acceptance	Rosenberg (1965)
Lee [39]	High school student (unclear)	F	321	+	Nursing	AEBI, AOBI, ASAA, BMI, CS needs, ideal BMI, ISAA	Rosenberg (1965)
Lee [40]	Undergraduate (unclear)	F	350	–	Fashion design	Apparel MB, appearance management cost, appearance satisfaction, BMI, cosmetics MB, hair care behavior, makeup MB, weight control behavior	Rosenberg (1965)
Lee and Park [41]	Undergraduate (22.0)	F	232	–	Business administration	ASAA, ISAA, media concern, physical appearance comparison	Rosenberg (1965)
Lee and Yang [42]	Adult (46.74 ± 11.31)	F	201	+	Nursing, beauty science	Age, appearance interest, appearance MB, CS interest, weight control intent	Rosenberg (1965)
Lee et al. [43]	Undergraduate (unclear)	M, F	287	–	Business administration	Care about appearance, Fashion pursuit, health pursuit, media exposure, self-monitoring level	Rosenberg (1965)
Son [44]	Undergraduate (20.62 ± 1.74)	F	161	–	Psychology	PS experience (yes), PS experience (no), PS satisfaction (yes), PS satisfaction (no)	Rosenberg (1965)
Suh and Jeong [45]	High school student and adult (28.47 ± 11.13)	F	355	–	Counseling, beauty science	Carefulness to choose beauty salon, carefulness to choose hairdresser, hair conditioner preference, hair management difficulties, hairdressing satisfaction	Rosenberg (1965)

Cal., calculation; AEBI, appearance evaluation in body image; AOBI, appearance orientation in body image; AS, aesthetic surgery; ASAA, awareness in sociocultural attitude toward appearance; BAS, body area satisfaction in body image; Cal., calculation; CA, clothing attitude; CS, cosmetic surgery; F, female; IBMI, body mass index; ISAA, internalization in sociocultural attitude toward appearance; M, male; MB, management behavior; PS, plastic surgery

Given the heterogeneity of the subjects, we calculated the overall effect sizes in the meta-analysis based on the random-effects model. The variables used to calculate correlations with self-esteem were grouped into six categories: appearance management intention, cosmetic surgery intention, sociocultural attitude, body satisfaction, body mass index (BMI), and stress. In cases where the analysis results were heterogeneous, we homogenized the studies using k-means clustering.

**Fig. 2:**
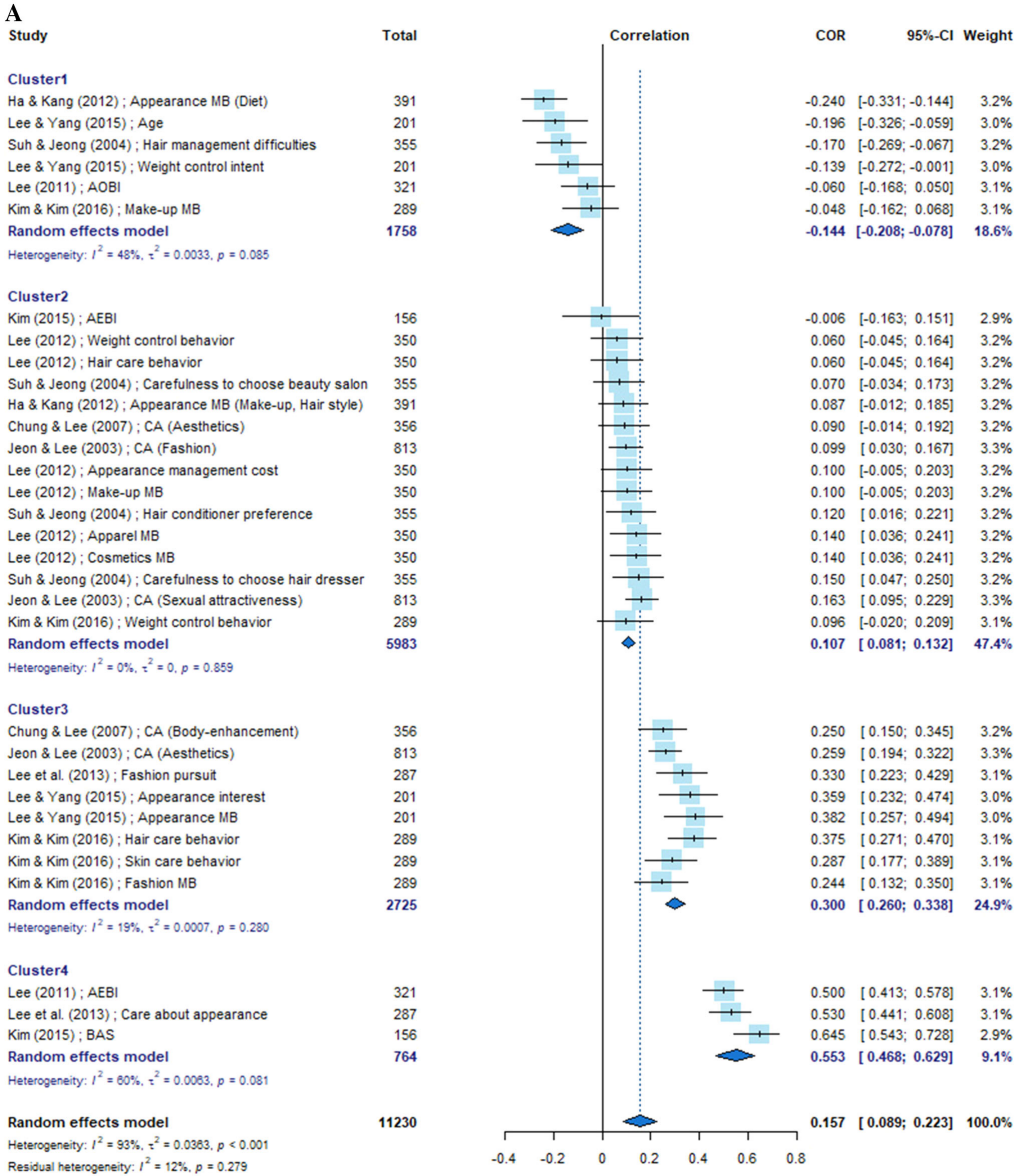

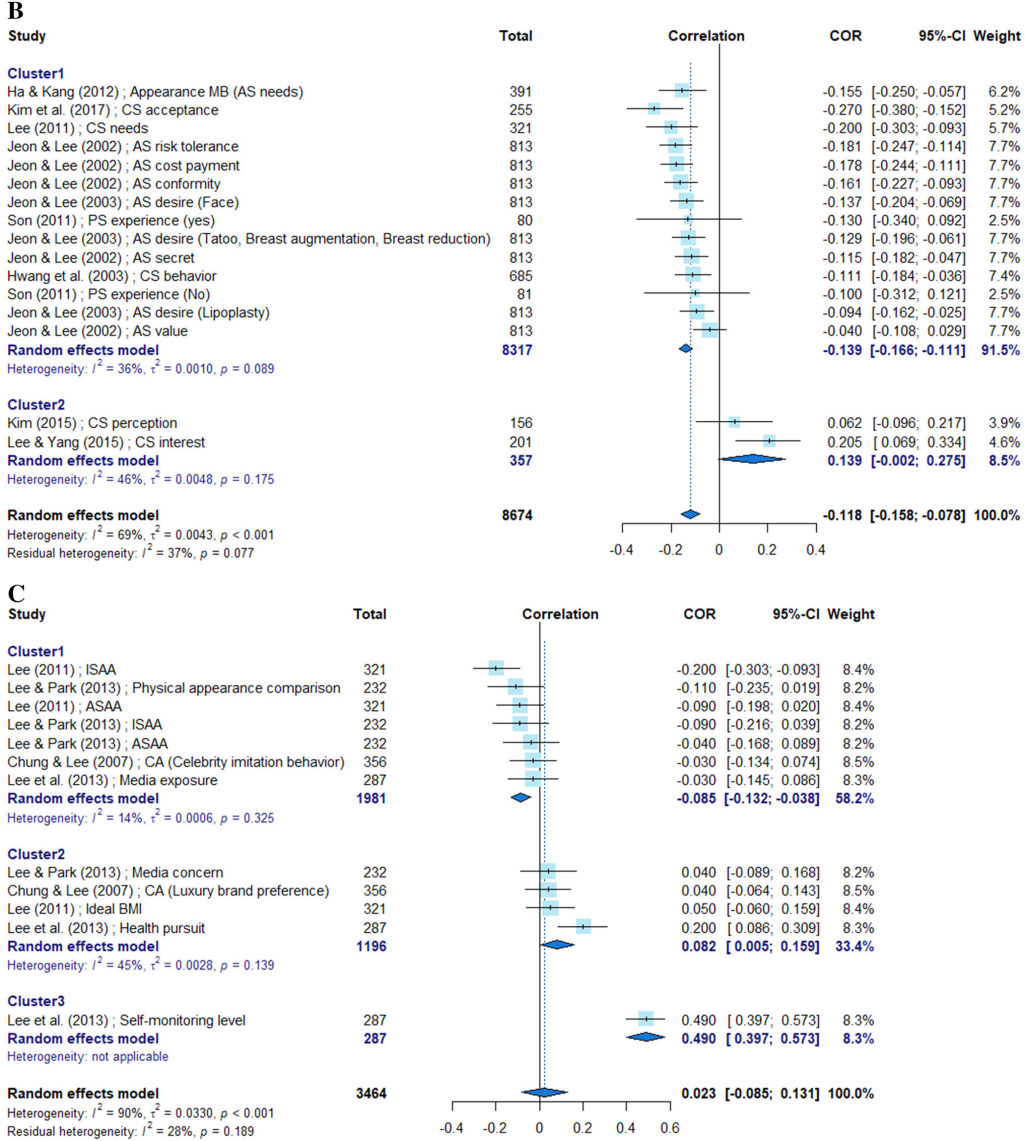

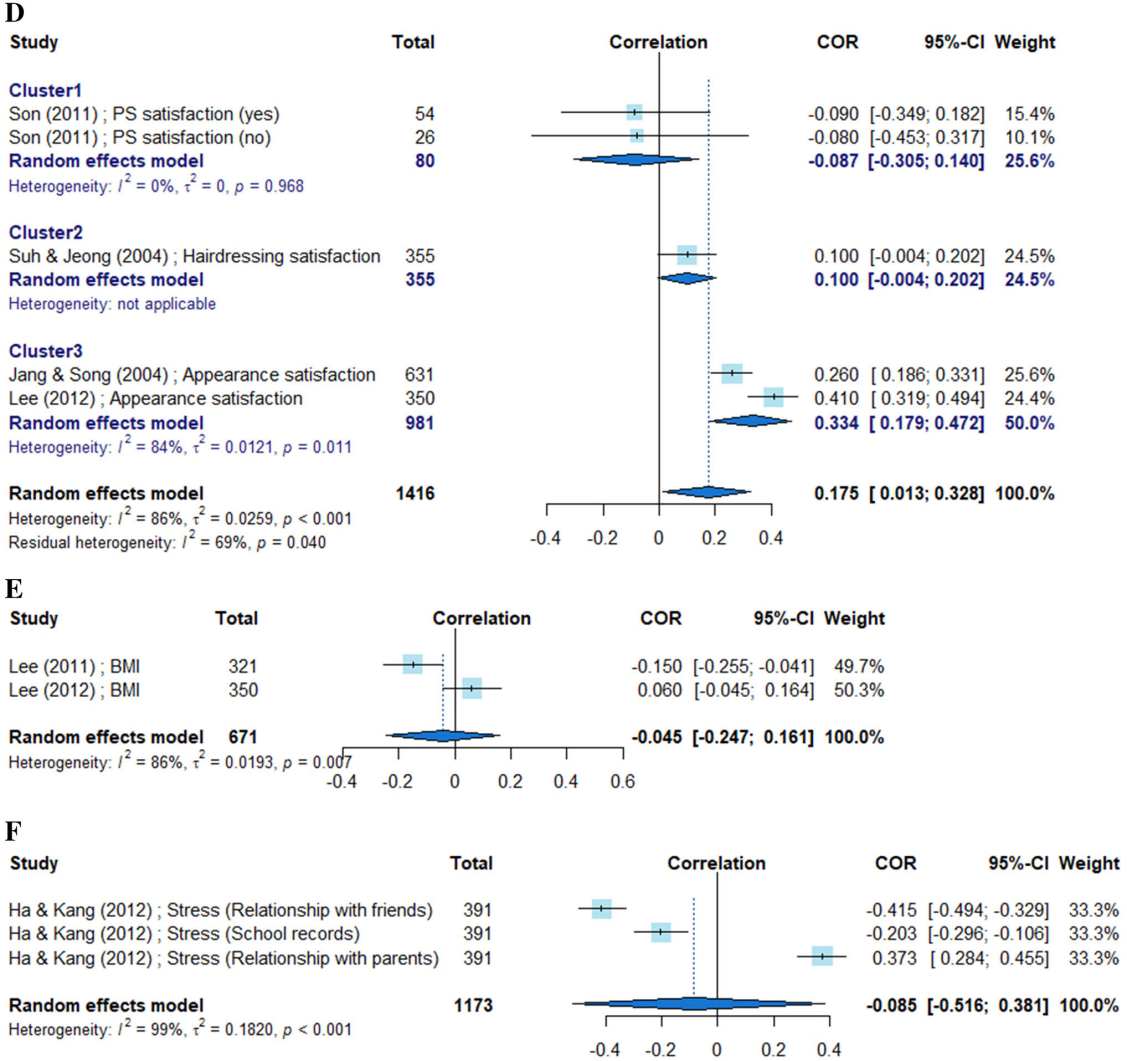
Forest plots of main variables

Ten studies contained 32 relevant variables pertaining to the category “appearance management intention;” their mean effect size was 0.157 in the meta-analysis based on the correlation coefficients. Because the effect sizes of individual variables could be grouped into four clusters, we arranged them in a forest plot according to the effect size level. Nine studies contained 16 relevant variables pertaining to the category “cosmetic surgery intention,” with a mean effect size of − 0.118. The effect sizes of individual variables were grouped into two clusters. Four studies dealt with 12 variables pertaining to the category “sociocultural attitude;” their effect sizes were grouped into three clusters with a mean effect size of 0.023. Four studies dealt with five variables pertaining to the category “body satisfaction;” their effect sizes were grouped into three clusters, with a mean effect size of 0.106. Two studies dealt with BMI-related variables, with a mean effect size of − 0.045. One study dealt with three stress-related variables, with a mean effect size of − 0.085 (Fig. 2).

In addition, influences on the overall effect size and contribution to heterogeneity in each category were examined, and suspicious variables were summarized (Table 2), because some studies may exert a particularly high influence on the overall results. The contribution of each study to the overall heterogeneity was measured by Cochran’s Q [47].

**Table 2:** The suspicious variables for meta-analysis

	Contribution to heterogeneity	Influence on overall result
Cosmetic surgery intention		
CS acceptance	4.932	0.155
AS value	7.613	0.820
Appearance management intention		
Appearance MB (diet)	3.732	1.071
AOBI	2.386	0.534
Makeup MB	2.784	0.540
CA (sexual attractiveness)	2.674	0.422
Sociocultural attitude		
Health pursuit	4.157	1.310
ISAA	4.377	0.848

## Discussion

To the best of our knowledge, this study is the first systematic literature review and meta-analysis of variables related to self-esteem in Korean studies to examine the relationship between cosmetic surgery and self-esteem. Korean studies have examined the relationship between cosmetic surgery and self-esteem using a wide variety of heterogeneous variables. For this meta-analysis, the variables were grouped into six categories based on consensus between the two researchers.

In the category “appearance management intention,” body area satisfaction in body images, appearance evaluation in body images, and care about appearance had the highest mean effect sizes. Notably, the body image subscales showed different levels of effect sizes, with the variables related to overall appearance showing higher effect size levels than the subscale variables, such as fashion, makeup, and hairstyle. One explanation for this finding may be that individual preferences come into play in the appearance management subscales. The effect size of cluster 4 containing variables pertaining to overall appearance was 0.553, which was rated high according to the criteria proposed by Cohen [48]. This result reflects the globally increasing acceptance of cosmetic surgery and the social atmosphere emphasizing the physical appearance [10, 15, 18, 21].

Among the variables pertaining to the category “cosmetic surgery intention,” the degree of interest showed the greatest effect size, whereas the experience and value of cosmetic surgery exhibited an insignificant relevance. This result supports findings of previous reports that cosmetic surgery does not influence self-esteem [10, 17]. Most studies yielded negative effect sizes, demonstrating that variables expressing the relationship between self-esteem and cosmetic surgery are inversely correlated. In particular, Lee and Yang [42], whose subjects were North Korean female defectors (mean age = 46.74 years), demonstrated characteristics that were different from those reported in other studies. This finding suggests that differences in sociocultural characteristics and systems are associated with varying degrees of interest in cosmetic surgery. The findings of study #3, whose subjects were college students with the same major (nursing), were likely to be subject-biased compared with those of other studies.

Among the variables pertaining to the category “sociocultural attitude,” self-monitoring showed the highest positive effect size. An ideal BMI, luxury brand preference, and media concerns were found to be statistically insignificant, supporting the findings of a previous study that media consumption was not correlated with self-esteem [19]. A similar result was obtained for the category “appearance management intention,” in which the sociocultural attitude subscales reflecting individual tastes had little relevance to self-esteem.

Among the variables pertaining to the category “body satisfaction,” satisfaction with overall physical appearance showed a higher effect size than satisfaction with hairdressing, and satisfaction with cosmetic surgery did not reach statistical significance. In the BMI category, only study #5 showed a negative effect size, demonstrating an inverse correlation with self-esteem. The subjects of this study were high school girls, reflecting the weight-control trend among Korean teenagers. In the stress category, one study stood out by focusing on the correlation between stress and self-esteem based on peer relationships, parental relationships, and academic achievement subscales. In particular, the effect size of peer relationships was relatively high (− 0.415), demonstrating the effect of peer influence during adolescence.

Social appearance evaluations affect self-esteem [16], and low appearance satisfaction can lead to lack of self-esteem [38]. In particular, Suissa’s description of cosmetic surgery addiction in Korea depicts a strong social pressure to meet commonly accepted standards of beauty for women of a marriageable age; however, the highly competitive nature often makes it insufficient to simply alter the physical appearance [49]. Moreover, in a randomized survey conducted in Korea, personal experience with cosmetic surgery had the greatest impact on the addictiveness of undergraduate women to cosmetic surgery [10]. A total of 44.8% of college students have been reported to experience their first cosmetic surgery in their teens, and overwhelmingly these subjects use mass media and the internet instead of consultation with experts to obtain information on cosmetic surgery [38]. Therefore, long-term studies on the impact of social media on cosmetic surgery are needed.

## Conclusion and Suggestions

The significance of this study is that we conducted a quantitative and integrative analysis of the variables related to self-esteem in Korean studies and examined the relationship between cosmetic surgery and self-esteem. Among the relevant variables categorized in this study, sociocultural attitude, BMI, and stress showed weak effect sizes, whereas appearance management intention, cosmetic surgery intention, and body satisfaction showed intermediate effect sizes. The appearance management intention category showed a strong effect on overall appearance satisfaction compared with that of the subordinate appearance variables according to individual preference. Cosmetic surgery is performed to satisfy subordinate appearance variables, but the intention appears to increase overall satisfaction with the appearance. The negative correlation between cosmetic surgery and intention suggests that cosmetic surgery helps increase self-esteem. Among the variables pertaining to the “sociocultural attitude” category, self-monitoring showed the highest positive effect size, whereas the effect size of body satisfaction was similar to that of appearance management intention. Further studies on BMI and stress are necessary, because the number of studies is insufficient to assess the overall effects. The methodology and findings of this study are expected to serve as basic data that will ensure more positive clinical outcomes. The overall characteristics of individuals seeking cosmetic surgery will be evaluated further as the number of related studies increases through follow-up studies with larger sample sizes.
